# Road Traffic Injuries, Trends, and Patterns: A Five-Year Retrospective Analysis Using Secondary Police Data in Nepal

**DOI:** 10.31729/jnma.v64i293.9290

**Published:** 2026-01-31

**Authors:** Raymond B C, Jeena Khadka, Gaurav Devkota, Pusp Raj Bhatt, Puspa Basnet, Chetan Bhatta

**Affiliations:** 1 Nepal Health Professional Council, Bansbari, Kathmandu, Nepal; 2 Australian Health Practitioner Regulation Agency, Melbourne, Victoria, Australia; 3 Nepal Public Health Association, Jwagal, Lalitpur, Nepal; 4 Nepal Injury Research Centre, KMC, Duwakot, Bhaktapur, NepalO; 5 Nepal Health Research Council, Ramshah Path, Kathmandu, Nepal; 6 Lambton College, Toronto, Ontario, Canada

**Keywords:** *automobiles*, *incidences*, *mortality*, *Nepal*, *road traffic accident*

## Abstract

**Introduction:**

Road traffic accidents are a major public health concern in Nepal, causing significant morbidity and mortality. The study goal was to determine the trends of road traffic injuries in Nepal from Fiscal Years 2020/21 to 2024/25 (mid-July 2020 - mid-July 2025).

**Methods:**

A descriptive retrospective study was conducted to analyze de-identified and pooled road traffic accident records from Nepal, following receipt of ethical clearance from the Nepal Health Research Council (ERB no. 607_2025). Data were analyzed using Microsoft Excel 2019.

**Results:**

The findings show that road traffic accidents have shown an apparent spike from FY 2020/21 to 2024/25, with both vehicle collisions and accident incidences ascended significantly. Reported vehicle crashes rose from 33135 in FY 2020/21 to 43165 in FY 2024/25, while total RTAs increased from 20640 to 28692 over the same period. RTAs surged in six provinces of Nepal. Two-wheelers, four-wheelers, and public transport vehicles accounted for the majority of incidents. Speeding 46398 (44.08%), mechanical failure 1173 (39.14%), potholes 685 (21.69%), pedestrian road crossings 5523 (79.55%), and unfavourable weather conditions like fog and mist 122 (26.23%) were major contributing factors. Although injuries increased significantly, fatality rates did not rise in same proportion.

**Conclusions:**

The results show that road traffic accidents are becoming more common in Nepal, especially involving motorbikes, four-wheelers, and public vehicles. Reducing accident rates and their effects may require increased road safety enforcement, better infrastructure, and awareness-raising initiatives.

## INTRODUCTION

Road traffic accidents (RTAs) are a serious global public health concern, killing an estimated 1.19 million people each year, and 20 to 50 million more suffer non-fatal injuries, many of which result in long-term disability.^1,2^ Economic and societal losses, with economies losing an estimated 2-7 % of their Gross Domestic Products (GDP).^3,4,5^

Nepal is experiencing an increase in RTAs due to rapid urbanization, more motorization, hard terrain, and inadequate enforcement of safety standards. The study provided national-level evidence of increased rates of crashes, particularly involving two-wheelers and four-wheelers in Nepal, establishing the context for the current study.^6^

Despite the growing public health concern about RTAs in Nepal, previous studies reported rising trends, predominantly affecting young individuals. Temporal trends, causative factors, and subnational analysis remain insufficiently explored. The purpose of the study is to examine the epidemiological features, trends, and contributing factors of RTAs in Nepal between FY2020/21 and FY2024/25.

## METHODS

This descriptive retrospective study utilized secondary, deidentified, and pooled RTA’s records from the Nepal Police Central Headquarters archive. The dataset comprised accident records reported to the Road Accident Information Management System (RA-IMS), a centralized electronic database maintained by the Nepal Police. The system is based on a standardized Accident Form (ARF) that is completed by traffic police officers at the scene of the crash and captures structured information on crash circumstances, involving all categories of road users, including road users; drivers, passengers, pedestrians, vehicles, environmental conditions, injury severity, and outcomes. All RTAs recorded in RA-IMS during the five-year study period with complete information on time, location, cause, vehicle type, outcome, and involving any category of road user from all provinces of Nepal were included. Records outside the study period, with missing critical details, duplicates, or unverified data not entered in RA-IMS, were excluded.

The study obtained written permission from the Nepal Police and the ethical review board, i.e., the Nepal Health Research Council (ERB no. 607_2025). National-level data were analyzed covering RTA’s data of all seven provinces of Nepal and spanning five years (fiscal year 2020/21 to 2024/25). The Metropolitan Traffic Police Division (MTPD) oversees traffic management in the Kathmandu Valley, which includes Kathmandu, Lalitpur, and Bhaktapur districts. Its jurisdiction extends to metropolitan and municipal areas, national and provincial highways, ring roads, and key arterial routes. Province Traffic Police Offices handle traffic laws outside of the Kathmandu Valley. The Koshi Province Traffic Police Office, situated in Itahari, serves all 14 districts in the province. The Madhesh Province Traffic Police Office in Pathlaiya oversees eight districts. The Bagmati Province Traffic Police Office, based in Ramnagar, Chitwan, has jurisdiction over ten districts (excluding the Kathmandu Valley districts). The Gandaki Province Traffic Police Office in Gagangauda, Pokhara, is in oversight of 11 districts. The Lumbini Province Traffic Police Office in Butwal serves 12 districts. The Karnali Province Traffic Police Office in Birendranagar oversees ten districts, while the Sudurpaschim Province Traffic Police Office in Attariya manages nine.^7^ These jurisdictions cover national and provincial highways, urban areas, intersections, arterial roadways, and communities throughout Nepal. For each accident, geographical location (province), number of vehicles involved, and injury severity (fatal, major, minor) were recorded. Demographic variables of victims and contributing risk factors for RTAs were also examined. Descriptive statistics were calculated using Microsoft Excel 2019.

## RESULTS

Analysis of reported RTA occurrences from the Nepal Police archive revealed RTAs from fiscal year 2020/21 to 2024/25. There were a total of 33135 vehicle collisions and 20640 RTAs in FY 2020/21, and 43165 vehicle collisions and 28692 RTAs in FY 2024/25. Two-wheeler vehicles accounted for vehicle collisions of 17087 (51.57%) in FY 2020/21 to 25142 (58.25%) in FY 2024/25. Car and jeep collisions numbered in FY 2020/21, 7,559 (22.81%) and 6,644 (15.39%) collisions in FY 2024/25. Three-wheeler vehicle collisions were reported in FY 2020/21, 899 (2.71%), and 2478 (5.74%) in FY 2024/25, as shown in Table 1. Between FY 2020/21 and FY 2024/25, data show counts of total injuries, severe injuries, minor injuries, and deaths due to RTAs. Serious male injuries was reported as 5197 (80.59%) in FY 2020/21 and 5254 (75.32%) in FY 2024/25. Serious female injuries were reported as 1251 (19.41%) in FY 2020/21 and 1722 (24.68%) in 2024/25. The male-to-female ratio of serious injuries was 3.43. Male minor injuries were reported as 14187 in FY 2020/21 and 27141 in FY 2024/25, with a male-to-female ratio of 1.91. Female minor injuries were reported as 4,413 in FY 2020/21 and 10,142 in FY 2024/25.

**Table 1:** Snapshot of Road Traffic Accidents (RTAs) in the Past five years (n=120393).

Classification	FY 2020/21	FY 2021/22	FY 2022/23	FY 2023/24	FY 2024/25
Total RTA	20640	24537	23597	22927	28692
Total Vehicle Collision	33135	39379	37393	35404	43165
Motorcycle	17087(51.57)	19974(50.72)	19511(52.18)	19984(56.45)	25142(58.25)
Jeep, Car	7559(22.81)	8975(22.79)	8432(22.55)	6837(19.31)	6644(15.39)
Truck, Tanker	2299(6.94)	2476(6.29)	2005(5.36)	1486(4.20)	1763(4.08)
Bus	1719(5.19)	2786(7.07)	2850(7.62)	2193(6.19)	1993(4.62)
Manually driven cart/cycle	1340(4.04)	1564(3.97)	1404(3.75)	1520(4.29)	2390(5.54)
Tractor	993(3.00)	1018(2.59)	825(2.21)	783(2.21)	1159(2.69)
Tempo (3 wheelers)	899(2.71)	1181(2.99)	1182(3.16)	1473(4.16)	2478(5.74)
Tipper	780(2.35)	772(1.96)	508(1.36)	466(1.32)	646(1.49)
Micro	234(0.71)	384(0.98)	477(1.28)	482(1.36)	654(1.52)
Dozer, loader, excavator	79(0.24)	73(0.19)	67(0.18)	73(0.21)	82(0.19)
Unknown	146(0.44)	176(0.45)	132(0.35)	107(0.30)	214(0.49)
Total Injured	25048	33004	30226	32156	44259
Serious Injury	6448	7282	5738	6160	6976
Male	5197(80.60)	5684(78.06)	4355(75.89)	4757(77.22)	5254(75.32)
Female	1251(19.40)	1598(21.94)	1383(24.11)	1403(22.78)	1722(24.68)
Minor Injury	18600	25722	24488	25996	37283
Male	14187(76.27)	19132(74.38)	18013(73.56)	18954(72.91)	27141(72.80)
Female	4413(23.73)	6590(25.62)	6475(26.44)	7042(27.09)	10142(27.20)
Deaths	2500	2883	2376	2369	2549
Male	2098(83.92)	2366(82.07)	1894(79.71)	1900(80.20)	2036(79.88)
Female	402(16.08)	517(17.93)	482(20.29)	469(19.80)	513(20.12)

Overall, the ratio of men to women with minor injuries was 2.80. RTA-related deaths were reported as 2,500 in FY 2020/21 and 2,549 in FY 2024/25. Male fatalities were reported as 2,098 in FY 2020/21 and 2,036 in FY 2024/25. Female fatalities were reported as 402 in FY 2020/21 and 513 in FY 2024/25. Overall, the mortality ratio of men to women was 4.32 (Table 1).

**Figure 1:**
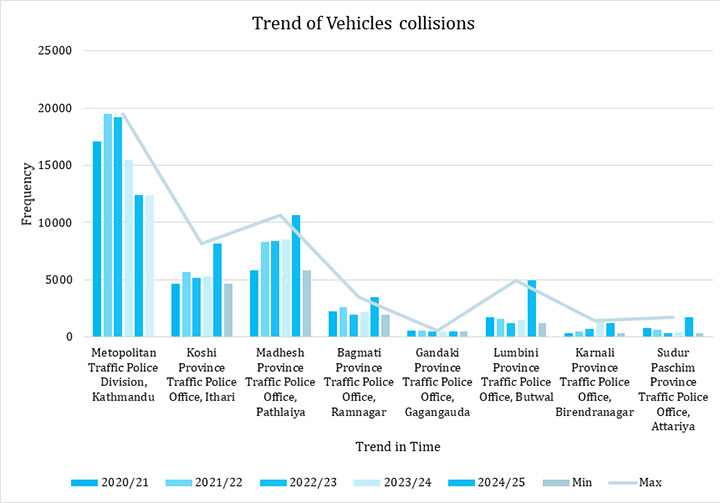
Trend of Vehicle Collisions Based on Metropolitan and Provinces (n=188476).

Traffic Police Office data gathered from seven provinces, as well as the Metropolitan Traffic Police Division (MTPD), revealed a variety of road traffic accident patterns. RTA incidents initially increased, showed fluctuations over the intermediate period before demonstrating a significant spike in the later years of the study period, FY 2020/21 to FY 2024/25, according to MTPD data. Particularly in FY 2024/25 and 2023/24, the ratio of RTA incidents outside the Kathmandu valley was 1.54 in Koshi, 1.25 in Madhesh, 1.59 in Bagmati, 3.22 in Lumbini, and 4.25 in Sudurpaschim provinces(Figure 1).

**Table 2:** Human Factors Contributing to Road Traffic Accidents in Nepal (n=112213).

Factors	n(%)
Driver induced RTA	105270(93.81)
Violation of traffic rules	4242(4.03)
Carelessly opening door	471(0.45)
Overtake	9922(9.43)
Speeding	46398(44.08)
Driver sleeping	1277(1.21)
Alcohol consumption	5533(5.26)
Use of mobile phone	688(0.65)
Authenticity not obtained	12959(12.31)
Others	23780(22.59)
Passenger induced RTA	6943(6.19)
Ascending in vehicle	111(1.59)
Descending from vehicle	233(3.36)
traveling by hanging	89(1.28)
Trip on the roof	21(0.30)
Showing body out of vehicle	358(5.16)
Crossing the road	5523(79.55)
Others	608(8.76)

**Table 3:** Vehicle factors contributing to RTA in Nepal (n=2997).

Factors	n(%)
Mechanical failure	1173(39.14)
Overload	281(9.38)
Old vehicles	164(5.47)
No brakes	1005(33.53)
Runaway tyre	91(3.04)
Flat tyres	141(4.70)
Due to vehicle lights	41(1.37)
Other	101(3.37)

**Table 4:** Environmental Factors Contributing to Road Traffic Accidents in Nepal (n=5183).

Factors	n(%)
Road condition induced RTA	3157(60.91)
Potholes	685(21.69)
Construction Materials	53(1.68)
Slippery (Oil or Other)	345(10.93)
Turn, Bend	516(16.35)
Uphill road	128(4.06)
Downhill road	307(9.72)
Narrow	326(10.33)
Dangerous Road	31(0.98)
Sewer/Drain	26(0.82)
Dilapidated road	88(2.78)
Road Construction	228(7.22)
Unpaved Road	301(9.53)
Gravel Road	29(0.92)
Paved Road	16(0.51)
Other	78(2.47)
Weather induced RTA	465(8.97)
Fog-Mist	122(26.23)
Storm	50(10.75)
Rain	95(20.43)
Flood	38(8.17)
Landslide	48(10.32)
Other	112(24.09)
Animals/livestock induced RTA	1561(30.11)
Animals	1561(100)

The study also found that among the human factors, driver-induced factors were speeding 4242 (44.08%), authenticity not obtained 12959 (12.31%), overtaking 9922 (9.43%), alcohol consumption 5533 (5.26%), and traffic offences 4242 (4.03%) as the most prevalent causes. Pedestrian-related reasons were equally troubling, with road crossings 5523 (79.55%) identified as the primary cause of passenger-involved accidents (Table 2).

In addition to human factors, vehicle factor-induced issues such as mechanical failure 1173 (39.14%) and braking malfunction 1005 (33.53%) have been recognized as leading causes of RTAs. Based on environmental factors, potholes 685 (21.69%) were the most common cause of road-related RTAs, followed by sharp twists or bends 516 (16.35%), narrow highways 326 (10.33%), and downhill roads 307 (9.72%), directly interconnected with the geography of the country (Table 3).

Similarly, adverse weather conditions, fog, and mist 122 (26.23% were identified as key determinants of RTAs. Rain 95 (20.43%) was found as another major weather-related component that contributes to accidents. Animals were also the factors leading to road traffic accidents in Nepal (Table 4).

## DISCUSSION

Globally, RTAs kill millions of people each year, making them a primary cause of death. The number of road traffic deaths in low- and middle-income countries (LMICs) is disturbingly high at 21 deaths per 100000 population, when compared to worldwide averages. Low- and middle-income countries (LMICs) account for over 60% of global car ownership, although they account for 92% of traffic-related fatalities worldwide. For the South-East Asia region, fatality rates are 16 per 100000 population, one of the second-highest in the region.^1^ According to recent research conducted in Nepal, mortality was 14 per 100000 people, which equates to approximately 4000 deaths per year due to RTAs^8^. Similarly, studies indicate Nepal suffers the highest burden of RTA, which constitutes a major public health and socioeconomic challenge. ^5,9,10^

The study found an increase in the trend of total road traffic accidents over the last five years, with a gradual rise in the incidence of vehicle crashes, indicating a 1.4 times increase in the number of RTAs compared to the initial years. Two-wheelers, tractors, tempos, micro, and human-powered/non-motorised vehicles saw the highest increase in incidences of vehicle collisions leading to accidents, outnumbering all other modes of transportation combined. In contrast, collisions between vehicles involving buses, cars, jeeps, and other heavy vehicles, crashes increased initially, then reduced, but their incidence rates remained higher than in the first FY 2020/21. Similarly, various studies done in the South and Southeast Asia region indicate that motorcycle and three-wheelers account for the highest number of road traffic accidents, and around 30% of total fatalities, and four-wheeled vehicles accounted for about 25% of fatalities.^11-14^

Based on official reports from the Ministry of Physical Infrastructure and Transport, RTA reports from 2024 continue to increase sharply in the country. In comparison to vehicles, the road has not increased as per the standards in the country report, indicating that about 26,132-kilometre-long roads have been created in the country.^15,16^ The current study’s review of five years of data indicates there have been more injuries and a decline in fatalities overall. The most suffered injuries were minor ones, 132089 (74.47%), followed by serious ones, 32604 (18.38%), and fatalities, 12677 (7.15%). Research conducted in the Emergency Department of Dhulikhel Hospital in Nepal on traumatic injuries, including RTAs, found that 27.6% of cases were of moderate severity, 8.1% were major injuries, and 63.1% were minor injuries.^17^ We discovered that the male mortality rate was higher than the female mortality rate, 203:47, which is approximately 4.32 times higher. Whereas the study done in Dhaka found that RTI fatalities in Dhaka, with male mortality 2.85 times greater than female, were related to increasing male road exposure.^18^ Since women often have less access to motor vehicles as well as outdoor activities, men’s greater mobility, vehicle access, and outdoor activities account for a significant portion of this disparity.^19-21^ Although road traffic injuries have increased, steady fatality rates highlight the need for government investment in better post-crash treatment, ambulance and trauma services, and trauma registries to improve outcomes.^14,22^

The current study found that speeding (44.08%), overtaking (9.43%), alcohol use (5.26%), and violation of traffic rules (4.03%) are contributing factors to driver-induced RTAs. Mechanical failure (39.14%), braking malfunctions (33.53%), and pedestrians crossing roads (80%) are also key contributing factors of RTIs. Data indicating the rise in the number of vehicles and the road conditions, the majority of which are potholes (22%), turning, bends (16%), and downhill roads (10%), could lead to vehicle crashes. Additionally, bad road conditions in hilly and mountainous places, particularly during the summer, rainy, and winter, enhance the danger of collision. These results are consistent with previous research, emphasising the complex nature of RTAs that have an immediate effect on drivers, vehicles, and pedestrians.^23-26^ Despite government commitments with SDG goals and the UN Decade of Action for Road Safety 2021-2030 to reduce the burden by at least 50% by 2030, road traffic accident-related mortality and morbidity in Nepal remain on the rise.^1,14,27,28^ This indicates the urgent need for more robust and faster governmental actions to reduce the increasing prevalence through strengthening traffic regulations (speeding, drink-driving, helmet and seat belt use), prioritizing road maintenance (pothole patching, road lights), promoting public awareness through targeted education and advertisement, and encouraging riders to exercise caution and manage speed in residential and accident-prone areas.

Our study has a few limitations. Its reliance on secondary police data raises the possibility of underreporting, incomplete records, and incorrect categorisation of traffic accidents, especially when there are minor injuries or unreported incidents. The analysis is limited to the variables that are accessible in the police database; the inclusion of “other” factors as contributing factors may make it more difficult to pinpoint the precise causes of accidents, and it is impossible to completely confirm the veracity of some factors that have been reported. Additionally, the study excluded hospital-based information since it is limited to certain regions and does not provide extensive nationwide coverage. These constraints may have influenced the completeness and accuracy of our analysis.

## CONCLUSION

The study discovered that road traffic accidents, vehicle collisions in provinces, fatalities, and injuries in Nepal had increased during the last five years. The most often involved vehicles were two-and four-wheelers, with males being disproportionately affected. Speeding, alcohol consumption, pedestrians crossing roads, brake failure, mechanical malfunctions, potholes, sharp turns/bends, fog and mist, and animals in particular were predominant contributors to accidents. These findings emphasize the importance of targeted licensing, mandatory use of reflective gear for riders in fog-prone provinces, reflective collars for animals, targeted road safety interventions, stricter traffic law enforcement for high-risk drivers, and context-specific preventive strategies for reducing the burden of RTAs across the country.

## Data Availability

The data are available from the corresponding author upon reasonable request.
